# Association between human papillomavirus and Epstein - Barr virus DNA and gene promoter methylation of *RB1* and *CDH1* in the cervical lesions: a transversal study

**DOI:** 10.1186/s13000-015-0283-3

**Published:** 2015-06-02

**Authors:** Thaís M McCormick, Nathalie HS Canedo, Yara L Furtado, Filomena A Silveira, Roberto J de Lima, Andréa DF Rosman, Gutemberg L Almeida Filho, Maria da Glória da C Carvalho

**Affiliations:** Laboratory of Molecular Pathology, Pathological Anatomy Service and Pathology Department, Clementino Fraga Filho University Hospital, Federal University of Rio de Janeiro - UFRJ, Rio de Janeiro, Brazil; Laboratory of Neuropathology, Pathological Anatomy Service and Pathology Department, Clementino Fraga Filho University Hospital, Federal University of Rio de Janeiro - UFRJ, Rio de Janeiro, Brazil; Gynecology Institute, Federal University of Rio de Janeiro - UFRJ, Rio de Janeiro, Brazil; Serviço de Anatomia Patológica, Subsolo - sala 09 (Citopatologia), Hospital Universitário Clementino Fraga Filho, UFRJ, Ilha do Fundão, Rio de Janeiro, RJ CEP 21941-913 Brazil

**Keywords:** Cervical cancer, HPV, EBV, Methylation, *RB1*,*CDH1*

## Abstract

**Background:**

Human papillomavirus (HPV) inactivates the *retinoblastoma 1* (*RB1*) gene by promoter methylation and reduces cellular E-cadherin expression by overexpression of DNA methyltransferase 1 (DNMT1). The Epstein-Barr virus (EBV) is an oncogenic virus that may be related to cervical carcinogenesis. In gastric cancer, it has been demonstrated that *E-cadherin* gene (*CDH1*) hypermethylation is associated with DNMT1 overexpression by EBV infection. Our aim was to analyze the gene promoter methylation frequency of *RB1* and *CDH1* and verify the association between that methylation frequency and HPV and EBV infection in cervical lesions.

**Methods:**

Sixty-five samples were obtained from cervical specimens: 15 normal cervices, 17 low-grade squamous intraepithelial lesions (LSIL), 15 high-grade squamous intraepithelial lesions (HSIL), and 18 cervical cancers. HPV and EBV DNA testing was performed by PCR, and the methylation status was verified by MSP.

**Results:**

HPV frequency was associated with cervical cancer cases (*p* = 0.005) but not EBV frequency (*p* = 0.732). Viral co-infection showed a statistically significant correlation with cancer (*p* = 0.027). No viral infection was detected in 33.3% (5/15) of controls. *RB1* methylated status was associated with cancer (*p* = 0.009) and HPV infection (*p* = 0.042). *CDH1* methylation was not associated with cancer (*p* = 0.181). Controls and LSIL samples did not show simultaneous methylation, while both genes were methylated in 27.8% (5/18) of cancer samples. In the presence of EBV, *CDH1* methylation was present in 27.8% (5/18) of cancer samples. Only cancer cases presented *RB1* promoter methylation in the presence of HPV and EBV (33.3%).

**Conclusions:**

The methylation status of both genes increased with disease progression. With EBV, *RB1* methylation was a tumor-associated event because only the cancer group presented methylated *RB1* with HPV infection. HPV infection was shown to be significantly correlated with cancer conditions. The global methylation frequency was higher when HPV was present, showing its epigenetic role in cervical carcinogenesis. Nevertheless, EBV seems to be a cofactor and needs to be further investigated.

**Virtual Slides:**

The virtual slide(s) for this article can be found here: http://www.diagnosticpathology.diagnomx.eu/vs/1159157579149317.

## Background

Cervical cancer represents an important public health problem, as it is the fourth most common type of carcinoma in women worldwide. This disease was responsible for 265,000 deaths in 2012, of which 87% occurred in developing countries [1]. According to the National Cancer Institute (INCA-Brazil), in general, its mortality/incidence ratio is 52% [2] and its survival rate is 70% [1].

Genital human papillomavirus (HPV) infection causes virtually all cervical cancer cases [3], and the factors correlated with development from the initial lesion to invasive carcinoma are poorly understood [4]. The 40 genotypes of genital HPVs can be classified as low-risk and high-risk based on their oncogenic ability [5]. Among the high-risk HPVs (HR-HPV), HPV16 is of major clinical importance, causing over 50% of cervical cancer cases [6].

Cervical carcinogenesis is a stepwise process in which genetic and epigenetic abnormalities are seen in regulatory genes. Epigenetic alterations may modify the expression of HPV genes or even host genes, leading to silencing of tumor suppressor genes (TSGs) by promoter hypermethylation [7].

The E7 HPV oncoprotein is essential for the host cell transformation and immortalization process [8]. It is known that this oncoprotein inactivates the *retinoblastoma 1* (*RB1*) gene by promoter methylation, which is essential in cervical tumorigenesis in humans [9]. E7 also has the ability to reduce cellular expression of E-cadherin, one of the major cell adhesion molecules, by overexpression of DNA methyltransferase 1 (DNMT1), an enzyme responsible for maintaining methylation patterns [10]. Thus, the cellular reduction of E-cadherin by gene promoter methylation may indicate a risk of local invasion and metastasis.

The Epstein-Barr virus (EBV), a member of the human herpes virus group, has been suggested as another oncogenic virus related to cervical carcinogenesis, as it is present in subclinical infection and invasive carcinoma of the cervix [11]. In gastric cancer, it has been demonstrated that hypermethylation of *CDH1*, which expresses the E-cadherin protein, is associated with DNMT1 overexpression by EBV infection [12]. However, until now, this association with cervical carcinogenesis has not been described.

The relationship between viruses and cancer is well established. However, the epigenetic pathways that determine the regression or persistence of infection and also the progression from precursor lesions to cancer are not clear. Therefore, our aim was to analyze the gene promoter methylation frequency of *RB1* and *CDH1* and to verify the association between that methylation frequency and HPV and EBV infection in cervical lesions as well as in normal cervical epithelia.

## Methods

### Samples

This transversal study was performed with samples obtained from cervical specimens of 65 women over the age of 18 who attended the Cervical Pathology Outpatient Clinic of the Gynecology Institute of the Federal University of Rio de Janeiro, Brazil, between July 2006 and July 2013, excluding only unavailable samples and/or those in poor condition. Because of those exclusion criteria, we worked with 65 samples.

The control samples were obtained from 15 cervical specimens with normal cytology and colposcopy, and 50 patients showing prior cytology with cervical lesions were classified by its cytological alterations as follows: 17 low-grade squamous intraepithelial lesions (LSILs), 15 high-grade squamous intraepithelial lesions (HSILs), and 18 cervical cancers.

Patients showing cytology with a diagnosis of HSIL or cancer were submitted to guided colposcopy biopsy, and cells were obtained from cervical brushings from patients with LSIL as well as from the control group.

The cervical smears and biopsies were taken as part of a routine screening program for cervical carcinoma. The cervical smears were collected with cervix brushes in phosphate-buffered saline, and the biopsies were obtained by cervical conization. All samples were analyzed in the Pathological Anatomy Laboratory from the same Gynecology Institute and were reviewed and classified by a certified pathologist.

This research was approved by ethical review boards from the Maternity School of the Federal University of Rio de Janeiro. Patients were asked to participate in the study, and informed consent was obtained before sample collection.

### DNA extraction

The DNA extraction of the biopsy samples and cervical brushings was performed as described by Lattario et al. (2008) [13]. Briefly, these samples were digested in 500 μL of solution containing 10 mM Tris-HCl, pH 7.5, 10 mM NaCl, 2% SDS, 10 mM EDTA, pH 8.0, and 15 μL 10 mg/mL proteinase K and incubated for 16 hours at 55°C, followed by phenol-chloroform (1:1) extraction. The DNA was precipitated using ethanol at -20°C for 16 hours, and then the samples were washed with 80% ethanol, re-suspended in 20 μL of water and stored at -20°C until use.

The DNA was isolated by previous PCR amplification with exon 5 p53 primers as an internal marker to ensure that the isolated DNA from samples and the following PCR were performed correctly, as described by Pestener et al. (1994) [14].

### Detection of HPV and EBV

HPV and EBV DNA testing was performed using PCR methods. Detection of HPV was performed with the MY09 e MY11 [15] consensus primers, which amplify a 450-bp fragment. To detect EBV, we used consensus primers TC67 and TC69 [16], whose product is 288 bp. Both amplifications were performed in a thermocycler. The protocol for HPV detection was as follows: 5 minutes of initial denaturing at 95°C; 35 cycles at 95°C, at 60°C and at 72°C for 1 minute at each temperature; and a final elongation step at 72°C for 10 minutes. The PCR for EBV detection was performed as follows: 1 minute of denaturing at 95°C; followed by 40 cycles of 1 minute at 94°C, 2 minutes at 55°C and 1 minute at 72°C; and a final elongation of 5 minutes at 72°C. The amplicons were stored at 4°C until time of use. HeLa and Raji cell lines were used as positive reaction controls for HPV and EBV, respectively. Samples containing just the reaction mixture without the template were analyzed as negative controls.

### Bisulfite treatment

The extracted genomic DNA underwent sodium bisulfite modification as described by Rosas et al. (2001) [17]. This method transforms the unmethylated cytosines into uracils and does not alter the methylated cytosines. Briefly, 1 μg of genomic DNA was diluted in 50 μL of distilled water and denatured in 0.2 M NaOH for 10 min at 37°C. The denatured DNA was then resuspended in 550 μL of freshly prepared solution containing 10 mM hydroquinone (Sigma, St. Louis, MO) and 3 M sodium bisulfite, pH 5.0 (Sigma), and incubated at 50°C. After 16 hours of incubation, the DNA samples were desalinated through a column (Wizard DNA Clean-Up System, Promega, Madison, WI), treated with 0.3 M NaOH for 15 min at room temperature and precipitated with ethanol. The bisulfite-modified genomic DNA was resuspended in 30 μL of distilled water and immediately used or stored at -20°C.

### PCR amplification of bisulfite-modified DNA

The sodium bisulfite modification was followed by methylation-specific PCR (MSP method). In this procedure, the treated DNA was used as a template for PCR amplification using specific primers for *RB1* [18] and *CDH1* [19], either methylated or modified unmethylated DNA. For PCR amplification, 4 μL of treated DNA was added to a 50-μL final volume of reaction mixture containing 1 X PCR buffer, dNTPs (1.25 mM each), primers (300 ng each per reaction), 1.5 mM MgCl_2_, and 1.25 units of *Taq* polymerase. Both amplifications were performed in a thermocycler. The protocol for the *RB1* analysis was as follows: 5 minutes of initial denaturing at 96°C; 35 cycles at 95°C, at 55°C and at 72°C for 1 minute at each temperature; followed by a final elongation step at 72°C for 7 minutes. The PCR protocol for *CDH1* analysis was performed as for HPV detection, as detailed previously. The primer pairs for MSP are shown in Table 1.

**Table 1:** **The primers pair sequences for MSP of** ***RB1*** **and** ***CDH1*** **and the amplicon sizes**

**Primer Pair**	**Sense 5′-3′**	**Antisense 5′-3′**	**Size (bp)**
RBM	GGGAGTTTCGCGGACGTGAC	ACGTCGAAACACGCCCCG	172
RBU	GGGAGTTTTGTGGATGTGAT	ACATCAAAACACACCCCA	172
ECM	GGTGAATTTTTAGTTAATTAGCGGTAC	CATAACTAACCGAAAACGCCG	204
ECU	GGTAGGTGAATTTTTAGTTAATTAGTGGTA	ACCCATAACTAACCAAAAACACCA	211

bp, base pair.

### Detection

The amplified PCR products were detected by 10% polyacrylamide gel stained with silver nitrate. The approximate amplified fragment sizes were visualized using the 100 Base Pair Ladder molecular weight marker (Pharmacia Biotech, USA).

### Statistical analysis

Statistical analysis was performed using GraphPad Software (GraphPad Software, Inc, USA). Fisher’s exact test was utilized, and the differences were considered to be statistically significant when the two-tailed P value < 0.05 (Confidence Interval = 95%).

## Results

### HPV and EBV detection

HPV DNA was detected in 26.6% (4/15) of control samples, while among the case samples, this frequency was 66.0% (33/50), specifically 64.4% (11/17) of LSIL cases, 53.3% (8/15) of HSIL cases and 77.8% (14/18) of cervical cancer samples. A significant association was observed concerning the presence of HPV and cervical cancer (*p* = 0.005). EBV DNA was found in 53.3% (8/15) of control group samples, 76.4% (13/17) of LSIL cases, 46.7% (7/15) of HSIL cases and 61.1% (11/18) of cervical cancer samples. However, the association between EBV infection and cervical cancer was not statistically significant (*p* = 0.732). Viral co-infection was found in all groups, with the lowest frequency in the control group (13.3%) and the highest in cancer cases (55.5%). These groups were compared, and a statistically significant association was found (*p* = 0.027). No virus infection was detected in one-third of the control samples (5/15), although in the case groups, this frequency was lower: 5.8% (1/17) in LSIL, 26.6% (4/15) in HSIL and 16.6% (3/18) in cancer samples. These results are summarized in Table 2.

**Table 2:** **Presence of HPV and EBV DNA in control, LSIL, HSIL and cervical cancer samples**

**Control/Lesion Type**	**HPV and EBV detection**			
	HPV, n (%)*	EBV, n (% )**	HPV/EBV co-infection, n (%)***	**No HPV and EBV infection, n (%)**
**Control (n = 15)**	4 (26.6)	8 (53.3)	2 (13.3)	5 (33.3)
**LSIL (n = 17)**	11 (64.7)	13 (76.4)	8 (47.1)	1 (5.8)
**HSIL (n = 15)**	8 (53.3)	7 (46.7)	4 (26.6)	4 (26.6)
**Cancer (n = 18)**	14 (77.8)	11 (61.1)	10 (55.5)	3 (16.6)
**Total (n = 65)**	37 (56.9)	39 (60.0)	24 (36.9)	13 (20.0)

* ***p*** **= 0.005 (Control** ***versus*** **Cancer).**

** ***p*** **= 0.732 (Control** ***versus*** **Cancer).**

*** ***p*** **= 0.027 (Control** ***versus*** **Cancer).**

LSIL, low-grade squamous intraepithelial lesions; HSIL, high-grade squamous intraepithelial lesions.

### *RB1* and *CDH1* methylation

**Figure 1:**
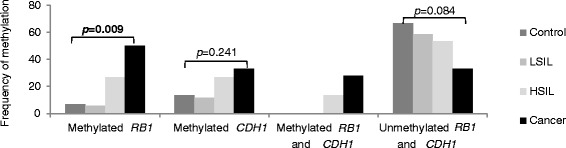
*RB1* and *CDH1* promoter methylation status. LSIL, low-grade squamous intraepithelial lesions; HSIL, high-grade squamous intraepithelial lesions.

The data concerning the promoter methylation status of *RB1* and *CDH1* are shown in Figure 1. The methylation status of both genes increased with disease progression, showing the lowest frequencies in the control (6.7% for *RB1* and 13.3% for *CDH1*) and LSIL samples (5.8% for *RB1* and 11.8% for *CDH1*) and the highest in cervical cancer samples (50.0% for *RB1* and 33.3% for *CDH1*). For *RB1*, its methylated status was strongly associated with cancer (*p* = 0.009). However, *CDH1* methylation was not shown to be associated with this cancer (*p* = 0.241). Controls and LSIL samples did not show co-methylation, while both genes were methylated in 27.8% of cancer samples (5/18). Otherwise, the unmethylated status decreased in frequency with disorder development, as 66.7% (10/15) of the control group and 33.3% (6/18) of cancer cases presented an unmethylated status in both genes. That decrease was not quite statistically significant (p = 0.084).

### Correlation between *RB1*/*CDH1* methylation status and HPV/EBV infection

In the presence of HPV, an increased methylation status of *RB1* was seen with developing pathological changes (up to 50.0%). In comparing LSIL and cancer samples, this condition was significantly associated with HPV infection (*p* = 0.042). The methylation status of *CDH1* increased in the presence of EBV (up to 45.5%); however, the difference was not statistically significant (*p* = 0.181). For the *RB1* methylation status, only cancer samples presented methylation in the presence of HPV and EBV (60.0%). For both genes, the global rate of methylation was higher when the virus was present: 32.4% for *RB1* in the presence of HPV vs. 15.3% without virus detection, and 25.0% for *CDH1* with HPV and EBV infection vs. 15.3% with no infection. These results are summarized in Table 3.

**Table 3:** **Comparison between** ***RB1*** **/** ***CDH1*** **methylation and HPV/EBV infection, reporting the percentages of methylated cases among HPV/EBV positive cases**

**Control/Lesion Type**	**Methylation Status**							
	***RB1***				***CDH1***			
	HPV+, n (%)*	**EBV+, n (%)**	**HPV and EBV+, n (%)**	**HPV and EBV-, n (%)**	**HPV+, n (%)**	EBV+, n (%)**	**HPV and EBV+, n (%)**	**HPV and EBV-, n (%)**
**Control (n = 15)**	0 (0.0)	0 (0.0)	0 (0.0)	1 (20.0)	1 (25.0)	0 (0.0)	0 (0.0)	1 (20.0)
**LSIL (n = 17)**	1 (9.1)	0 (0.0)	0 (0.0)	0 (0.0)	0 (0.0)	2 (15.3)	0 (0.0)	0 (0.0)
**HSIL (n = 15)**	4 (50.0)	0 (0.0)	0 (0.0)	0 (0.0)	4 (50.0)	2 (28.6)	2 (50.0)	0 (0.0)
**Cancer (n = 18)**	7 (50.0)	7 (63.6)	6 (60.0)	1 (33.3)	4 (28.6)	5 (45.5)	4 (40.0)	1 (33.3)
**Total (n = 65)**	12 (32.4)	7 (17.9)	6 (25.0)	2 (15.3)	9 (24.3)	9 (23.1)	6 (25.0)	2 (15.3)

* ***p*** **= 0.0421 (LSIL** ***versus*** **Cancer).**

** ***p*** **= 0.1819 (LSIL** ***versus*** **Cancer).**

+, presence; -, absence; LSIL, low-grade squamous intraepithelial lesions; HSIL, high-grade squamous intraepithelial lesions.

## Discussion

The presence of abnormal DNA methylation may represent a tool for detecting potential biomarkers with important roles in cervical carcinogenesis. Furthermore, HPV/EBV infection should improve these findings because it is correlated with worse clinical presentation [20].

In a systematic review of the literature, Wentzensen et al. (2009) [20] reported that *CDH1* was one of the most analyzed genes for methylation in cervical cancer. Nevertheless, a largely variable methylation frequency for both cancer and normal tissue was found among the studies. Feng et al. (2005) [21] reported the relevance of methylation in pre-cancerous lesions. Our study investigated the frequency of *RB1* and *CDH1* methylation in samples presenting all cervical carcinogenesis lesion stages and in normal tissue, in addition to reporting the correlation between this molecular event and HPV infection.

We detected HPV with a higher frequency among the case samples (66.0%) than the control (26.6%) in which 77.8% of the cancer group samples presented HPV. We found a very statistically significant association between HPV infection and cervical cancer (*p* = 0.005), as corroborated by the literature. In spite of that proved correlation, this infection alone is probably not sufficient to develop the disease. On the other hand, the samples in which HPV was not detected also presented cervical lesions. This molecular event was explained by Han et al. (2006) [22] and Sotlar et al. (2004) [23]. These studies show the existence of a gene deletion process in region L1 of HPV. This deletion occurs when HPV DNA integrates into the epithelial regions, and it has been described that it occurs in approximately 30% of the cases of positive cervical cancer samples. As the L1 region is the target of the primers applied in the current method for HPV detection, the absence of HPV detection in cancer cases could be explained by its deletion. Additionally, studies [24-28] about the Brazilian prevalence of HPV show that it is lower than in data from Walboomers et al. (1999) [29], which may be explained by the occurrence of the integration events affecting L1 sequences

EBV infection was found in a series of cases and control groups in which the rate of detection frequency was not considered statistically significant (p = 0.732). This finding indicates that EBV cannot be responsible for cervical cancer progression alone. Nevertheless, cancer was revealed to be associated with HPV and EBV coinfection (p = 0.027), showing its possible role as a cofactor in cervical cancer progression. Some preliminary studies found similar results (Nichols et al., 2011 [30]; Ekalaksananan et. al, 2011 [31]), indicating an association between co-infection with these viruses and cervical cancer progression. In a recent study, Khenchouche et al. (2013) [32] also highlighted the importance of this co-infection for cervical cancer progression, adding that it could be considered as a bad prognosis for this type of cancer.

In our study, the methylation status of both genes, RB1 and CDH1, increased with the severity of the cervical lesion, suggesting that the frequency of methylation was higher in cancers (50.0% for RB1 and 33.3% for CDH1) than in the other lesions and controls (6.7% for RB1 and 13.3% for CDH1 in controls). These data for CDH1 are consistent with findings in the literature (Chen et al., 2003 [33]; Dong et al., 2001 [34]; Narayan et al., 2003 [35]; Attaleb et al., 2009 [36]), which showed that CDH1 was methylated in less than 50% of cervical cancer samples, indicating that partial promoter methylation of the CDH1 can down-regulate the gene expression. Despite this, our data revealed that the association between CDH1 methylation and cancer was not significant (p = 0.241). Few studies have addressed RB1 methylation and its correlation with cervical carcinogenesis, and this issue needs to be further investigated. However, our results showed an expressive correlation between RB1 methylation and cancer samples (p = 0.009). It might be potentially used as a valuable marker for tumor diagnosis.

Only HSIL and the cancer group presented methylation in both genes simultaneously (13.3% and 27.8%, respectively), and these groups had the lowest frequencies for the unmethylated status in both genes (53.3% and 33.3%, respectively). Our findings also agree with those of Narayan et al. (2003) [35], who analyzed the methylation status of a group of genes in cervical carcinogenesis, including *RB1* and *CDH1*, and concluded that global promoter methylation was higher in more advanced stages of the disease. However, they did not find promoter methylation in the *RB1* gene. Our results indicate that these differences may be involved in the disease progression.

We also analyzed the potential association between *RB1*/*CDH1* methylation status and HPV/EBV infection. Our findings showed an important increase in the methylation status of both genes, *RB1* and *CDH1*, with a pathological change seen with HPV/EBV infection. It is important to note that the methylated status of *RB1* was considered to be associated with HPV infection (*p* = 0.042), revealing an important role for HPV in cervical cancer epigenetics.

The elucidation of the molecular relationships between viral and host proteins and their epigenetic modifications could improve the process of cervical cancer screening. Indeed, the detection of possible biomarkers and cofactors for the possibility of cervical cancer could allow for molecular differentiation between initial and precursor lesions.

## Conclusions

In conclusion, our results showed that the methylation status of both genes increased with disease progression, revealing a significant correlation between *RB1* methylation and cervical cancer. With EBV, the methylation of *RB1* was a tumor-associated event, where only the cancer group presented *RB1* methylation in the presence of this virus. HPV infection and cancer progression were significantly associated. The global frequency of methylation was higher when HPV was present, showing its important role in this epigenetic mechanism in cervical carcinogenesis. Nevertheless, EBV seems to have a cofactor role in this process, which needs to be further investigated.
